# Fly ash/Kaolin based geopolymer green concretes and their mechanical properties

**DOI:** 10.1016/j.dib.2015.10.029

**Published:** 2015-11-07

**Authors:** F.N. Okoye, J. Durgaprasad, N.B. Singh

**Affiliations:** a Department of Civil Engineering, Sharda University, Greater Noida, India; b Research and Technology Development Centre, Sharda University, Greater Noida, India

**Keywords:** Geopolymer, Cement, Concrete, Kaolin, Fly ash, Compressive strength

## Abstract

Geopolymer concrete mixes were cast using fly ash, kaolin, sodium hydroxide, potassium hydroxide, sodium silicate and aggregates. Portland cement concrete (M30) was used as a reference sample. The effect of silica fume, temperature (40 °C, 60 °C, 80 °C, 100 °C and 120 °C), sodium and potassium hydroxides and different superplasticizers on the compressive strength are reported [1]. Maximum strength was found at 100 °C and 14 M alkali solution [1].

## 1 Specifications Table

**Table:** 

Subject area	Materials Science
More specific subject area	Construction material
Type of data	Table, image, text file, graph, figure
How data was acquired	SEM, Strength measuring instrument
Data format	Raw, filtered, analyzed, etc
Experimental factors	Samples were left in oven at room temperature untill the day of testing.Curing of the samples were made at 100 °C.
Experimental features	Aggregates and binder were mixed dry in a drum mixer for 3 min, alkali added and mixing continued mixture becomes homogeneous.Compressive strengths under different conditions were measured
Data source location	Sharda University, Greater Noida, India.
Data accessibility	Data in this article.

## 2 **V**alue of the data

• The waste materials like fly ash and kaolin after reaction with alkali hydroxides/sodium silicate produced geopolymer green concrete.
• Mechanical properties of geopolymer concretes in the presence of silica fume are increased.
• The geopolymer concretes may be a suitable option for OPC concrete and this report will serve as a reference material for other researchers wishing to investigate the alternative building materials for construction industry.

## 3 Data

Fly ash/Kaolin based geopolymer concrete in the presence of different concentration of silica fume are made. Data on mechanical strength are presented.

## 4 Materials and methods

### 4.1 Materials

Commercially available kaolin and the fly ash obtained from National Power Station, Dadri, Uttar Pradesh, India, were used during the experiments. OPC-43 was used for making OPC concrete for comparing the compressive strengths of geopolymer concretes. Coarse aggregates of sizes 20 mm and 10 mm and river sand as fine aggregate were used. Sieve analyses were performed to determine the particle size distribution as per BS 812, Part1, 1975. Distilled water was used in all the experiments. Naphthalene sulphonate(N.S), Malamine-formaldehyde (GN-51) and Polycarboxylate ester (Chryso-730) based superplasticizers were used as admixtures. The alkali activators used were solutions of sodium hydroxide, potassium hydroxide and sodium silicate.

### 4.2 Preparation of alkalies

Solutions of sodium and potassium hydroxides (14 M each) were prepared separately. The solutions prepared were left for 24 h before mixing with sodium silicate. The mixtures of sodium hydroxide/potassium hydroxide and sodium silicate solutions were left for one day and then used for geopolymerisation process.

### 4.3 Mix proportion of geopolymer concrete

The geopolymer concrete was prepared by conventional method as OPC concrete. Since the density of geopolymer concrete is almost equal to that of OPC concrete (2400 kg/m^3^), aggregates occupy 75–80% by mass in geopolymer concretes. In the present mix design of geopolymer concrete, coarse and fine aggregates were taken as 77% by mass of the entire mixture. Fine aggregates were 30% by mass of the total aggregates. For maximum strength NaOH/KOH concentration was taken as 14 M [1]. Taking into consideration the workability, the ratio of sodium silicate to sodium hydroxide solution was kept 2.5 [1]. To improve the workability of fresh geopolymer mix, superplasticizer was used in all the mixes. In order to compare the effectiveness of different superplasticizers on compressive strength of geopolymer concrete, different doses of superplasticizers (Naphthalene sulphonate, Malamine-formaldehyde and Polycarboxylate ester based superplasticizers) were added separately to Mix 4 and compressive strength was measured. Six mixes were made. Four mixes Mix1, Mix2, Mix3 and Mix4 of geopolymer concretes using NaOH were prepared. Fifth Mix designated as Mix5 was also prepared by using KOH (14 M) instead of NaOH. Mixes with silica fume were also designed. A control mix with Portland cement (M30) was also prepared. The detailed mix design of geopolymer concrete mixes is given in Table 1.

### 4.4 Casting of geopolymer concrete mixes

The conventional technique used in OPC concrete was adopted [2], [3]. First fine and coarse aggregates were saturated surface dry (SSD) and then mixed together in 600 mm x 900 mm mixing pan for about 3 min. The alkali solution was mixed with superplasticizer and then added to the dry materials and mixing continued for 2 min. The whole mixture was then transferred into a tilting type drum concrete mixer and mixing continued for 3–5 min. The fresh geopolymer concrete formed pellets when homogeneously mixed in a drum concrete mixer and were very stiff in consistency as far as workability was concerned; however, adequate compaction was achieved. The mixture was cast in a 100 mm x 100 mm steel mould in three layers, and each layer given 60 strokes with 20 mm compacting rod. Eight cubes were cast for each mix beside the trial mixes. The cast samples were left in the laboratory at room temperature for 48 h.

### 4.5 Curing of geopolymer concrete

**Fig. 1:**
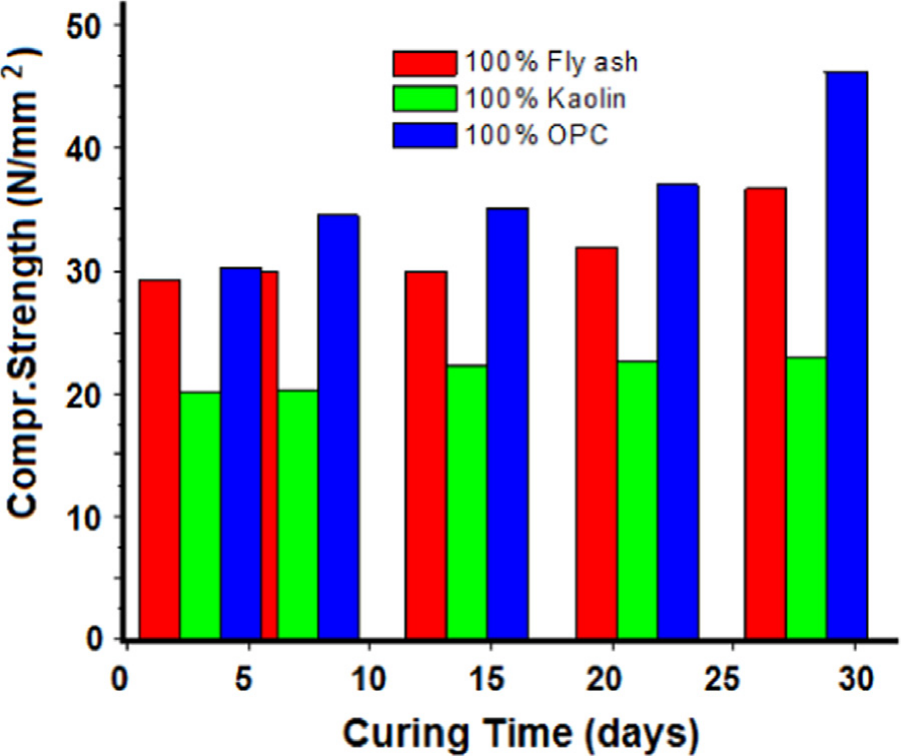
Variation of compressive strength with time.

The process of polymerisation requires high temperature and in order to know the optimum curing temperature. Mix4 after demoulding was heated at temperatures 40 °C, 60 °C, 80 °C, 100 °C and 120 °C for 72 h to know the optimum temperature (100 °C) for maximum compressive strength. After demoulding, all the Mixes were transferred in the oven for heat curing at 100 °C for 72 h. The samples were then left at room temperature after curing until the day of testing. The compressive strengths of the cubes with 100 wt% fly ash, 100 wt% kaolin and 100 wt% OPC was determined after 3 days, 7 days, 14 days, 21 days and 28 days (Fig. 1). Lower values of compressive strength of geopolymer concrete are due to presence of voids, which should be minimised during casting.

### 4.6 Effect of KOH on compressive strength

**Fig. 2:**
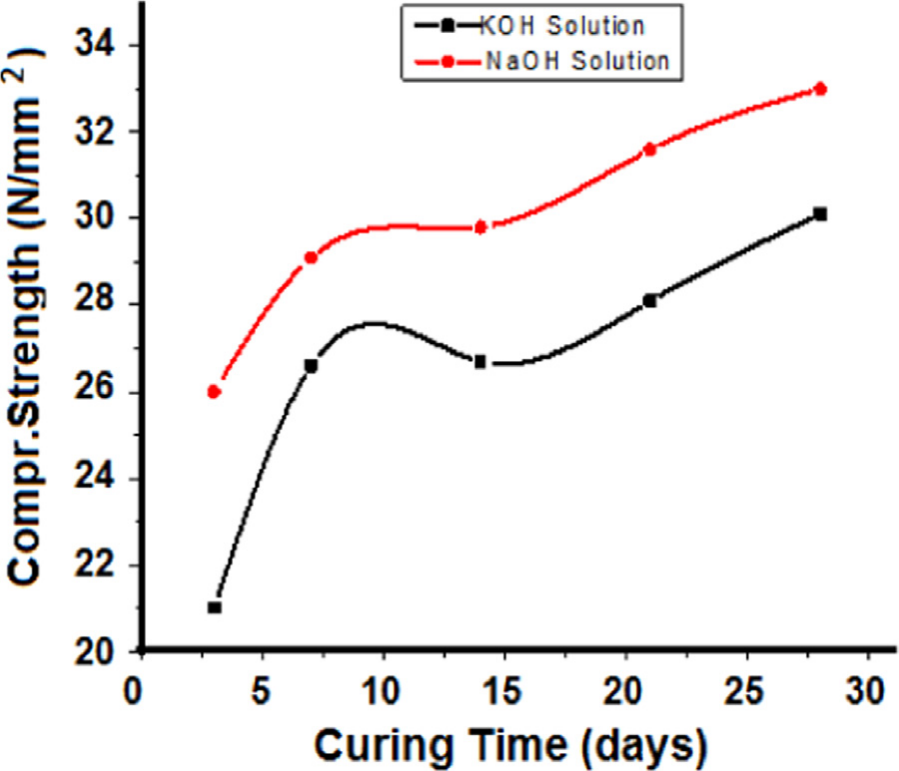
Variation of compressive strength with time in presence of NaOH and KOH.

Mix 5 given in Table 1 was prepared by using 14 M KOH in place of 14 M NaOH and the compressive strength was determined at different intervals of time as in the presence of NaOH. The compressive strength in the presence of NaOH and KOH was determined at different intervals of time and compared (Fig. 2). NaOH performed better as it is reported that in the presence of NaOH more monomers of silicates and aluminates are formed [4], [5].

### 4.7 Effect of different superplasticizers on compressive strength of Mix4

**Fig. 3:**
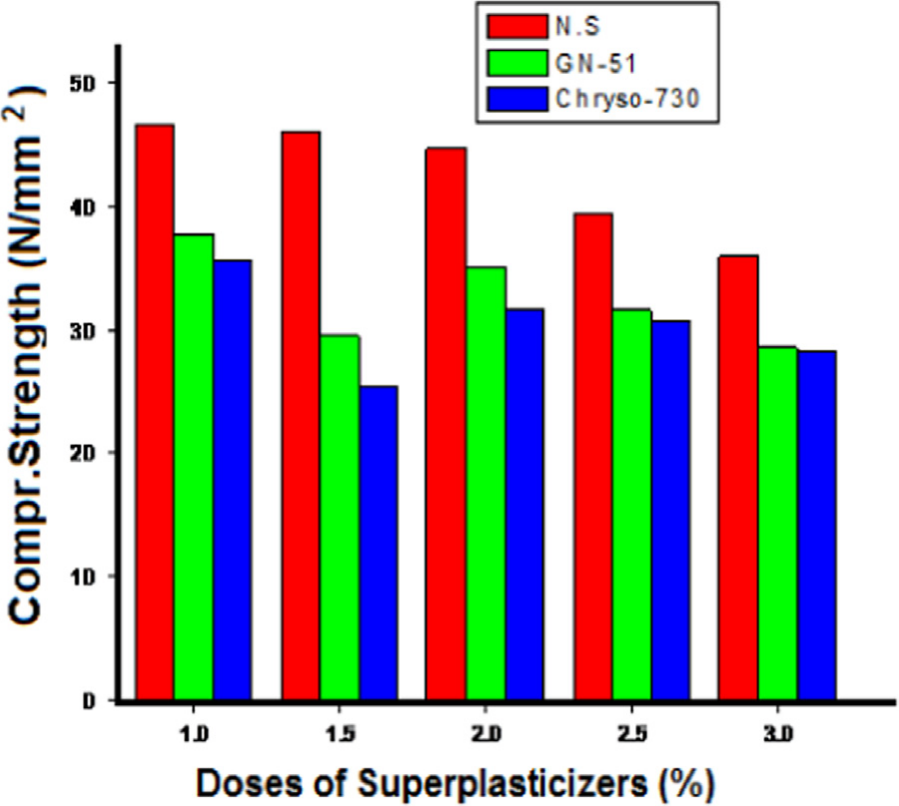
Effect of different superplasticizers on Compressive strength of geopolymer concrete (Mix4).

Variation of compressive strength with time in presence of different superplasticizers is shown in Fig. 3. 1.0 wt% naphthalene based superplasticizer gave highest strength and strength decreased at higher doses of the superplasticizers.

### 4.8 Compressive strengths of Fly ash and Kaolin based-geopolymer concrete

**Fig. 4:**
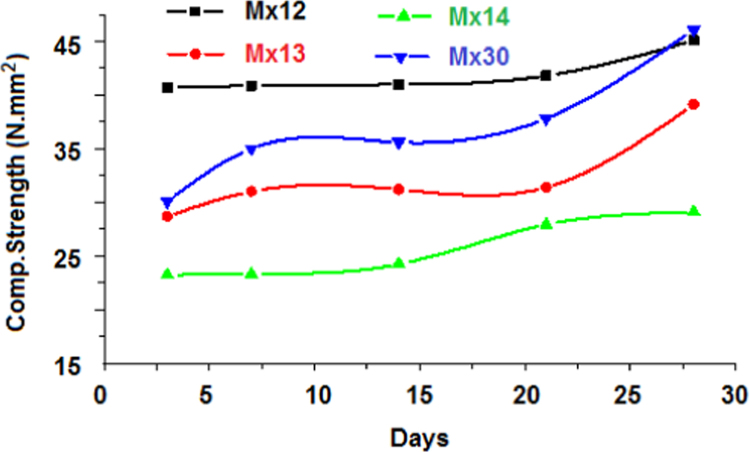
Variation of compressive strength with time in presence of silica fume.

Variations of compressive strength of Fly ash and Kaolin based-geopolymer concretes in presence of silica fume are given in Table 2 and Fig. 4. The geopolymer concrete containing silica fume gave higher compressive strength.

## Figures and Tables

**Table 1:** Mix proportion of geopolymer concrete.

Mix no.	Quantity of Ingredients (kg/m^3^)											
Coarse aggregate		Fine sand	Fly ash	Kaolin	OPC	SF	NaOH (14 M)	KOH (14 M)	SS	ALK/Binder	W/S
20 mm	10 mm
Mix1	862	431	554	388	0	0	0	45	−	113	0.4	0.2
Mix2	862	431	554	0	388	0	0	45	−	113	0.4	0.2
Mix3	862	431	554	349.2	38.8	0	0	45	−	113	0.4	0.2
Mix4	862	431	554	194	194	0	0	45	−	113	0.4	0.2
Mix5	862	431	554	194	194	0	0	−	45	113	0.4	0.2
Mix6	862	431	554	194	155	0	39	45	−	113	0.4	0.2
Mix7	862	431	554	116	233	0	39	45	−	113	0.4	0.2
Mix8	862	431	554	136	233	0	19	45	−	113	0.4	0.2
Mix12	862	431	554	194	155	0	39	45	−	113	0.4	0.2
Mix13	862	431	554	116	233	0	39	45	−	113	0.4	0.2
Mix14	862	431	554	136	233	0	19	45	−	113	0.4	0.2
M30	862	431	554	0	0	388	NA	−	NA	NA	NA	0.2

GP-12=50%FA+10%S.F+40%K GP-13=30%FA+10%S.F+60%K

GP-14=35%FA+5%S.F +60%K

FA-Fly ash, SS-Sodium silicate, ALK-Alkaline, W/S-Water/Solid ratio.

**Table 2:** Compressive strength of Fly ash based geopolymer concrete blended with Kaolin and silica fume.

Mix	Compressive strength (N/mm^2^)				
3d	7d	14d	21d	28d
Mix12	40.7	40.9	41.0	41.8	45.1
Mix13	28.7	31.0	31.2	31.4	39.1
Mix14	23.2	23.3	24.2	27.9	29.1
M30	30.1	35.0	35.6	37.8	46.1
