# Supplementary material for: Fly ash/Kaolin based geopolymer green concretes and their mechanical properties
Source: Data Brief. 2015 Nov 7;5:739–44. doi: 10.1016/j.dib.2015.10.029 (PMC4659805; doi:10.1016/j.dib.2015.10.029)

**Conflict of Interest**

We do not have any conflict of interest for the following paper.

**Fly ash/Kaolin based Geopolymer Green Concretes and their Mechanical properties**

**Authors:** F.N.Okoye^1^, J.Durgaprasad^1^ and N.B.Singh^2*^

**^1^**Department of Civil Engineering, Sharda University, Greater Noida, India

^2^ Research and Technology Development Centre, Sharda University, Greater Noida, India


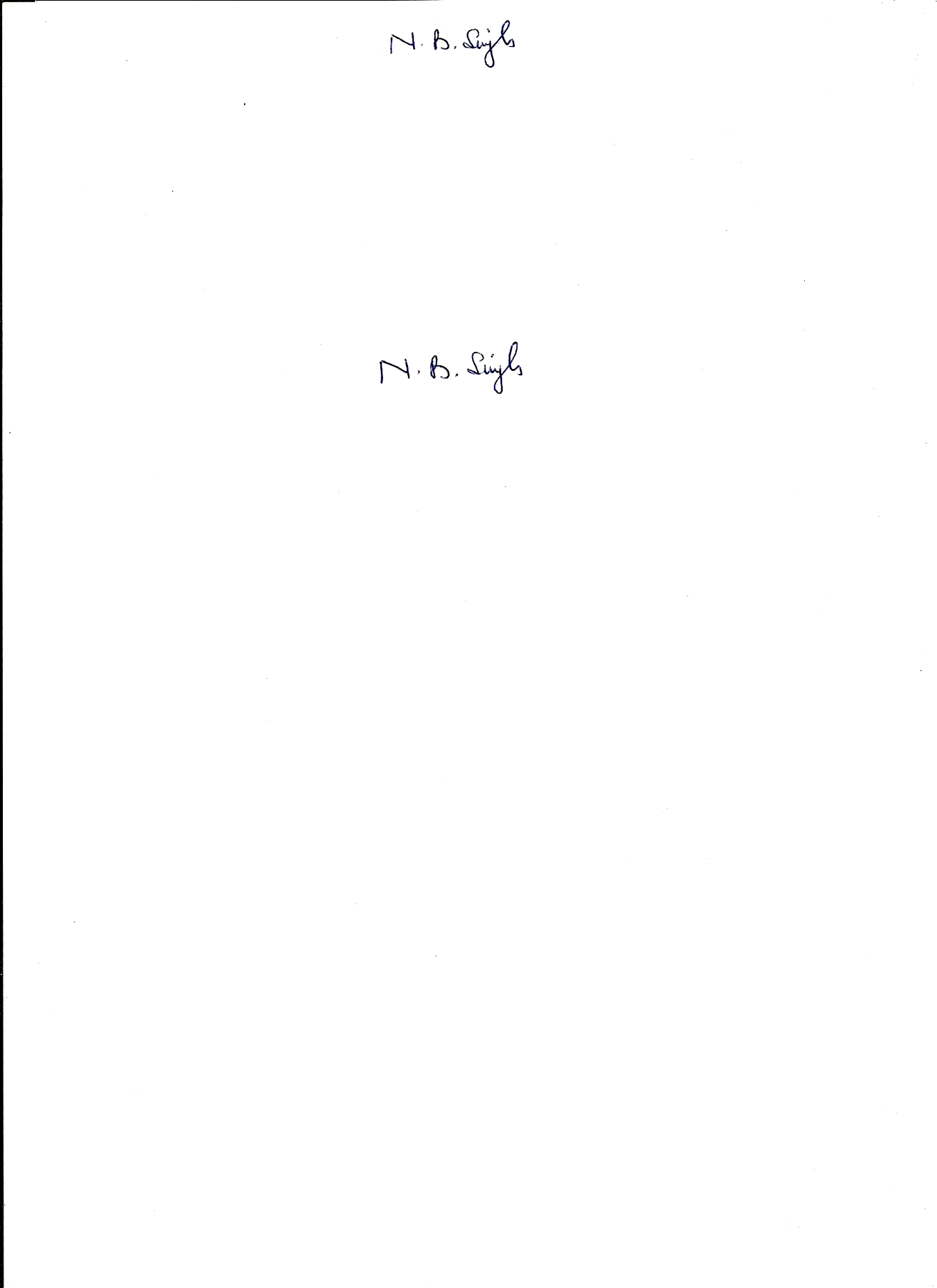

Supplement: Supplementary file 1 — Supplementary material [file mmc1.doc]
